# Overexpression of *KCNJ3* gene splice variants affects vital parameters of the malignant breast cancer cell line MCF-7 in an opposing manner

**DOI:** 10.1186/s12885-016-2664-8

**Published:** 2016-08-12

**Authors:** S. Rezania, S. Kammerer, C. Li, B. Steinecker-Frohnwieser, A. Gorischek, T. T. J. DeVaney, S. Verheyen, C. A. Passegger, N. Ghaffari Tabrizi-Wizsy, H. Hackl, D. Platzer, A. H. Zarnani, E. Malle, S. W. Jahn, T. Bauernhofer, W. Schreibmayer

**Affiliations:** 1 Institute of Biophysics, Molecular Physiology Group, Medical University of Graz, Harrachgasse 21/4, Graz, Austria; 2 Institute of Pathophysiology and Immunology, SFL Chicken CAM Laboratory, Medical University of Graz, Graz, Austria; 3 Division of Bioinformatics, Biocenter, Medical University of Innsbruck, Innsbruck, Austria; 4 Nanobiotechnology Research Center, Avicenna Research Institute, ACECR, Tehran, Iran; 5 Institute of Molecular Biology and Biochemistry, Medical University of Graz, Graz, Austria; 6 Institute of Pathology, Medical University of Graz, Graz, Austria; 7 Division of Oncology, Department of Internal Medicine, Medical University of Graz, Graz, Austria; 8 Research Unit on Ion Channels and Cancer Biology, Medical University of Graz, Graz, Austria; 9 Present address: Institute of Human Genetics, Medical University of Graz, Graz, Austria

**Keywords:** *KCNJ3*, Breast cancer, GIRK1, MCF-7, Splice variant

## Abstract

**Background:**

Overexpression the *KCNJ3*, a gene that encodes subunit 1 of G-protein activated inwardly rectifying K^+^ channel (GIRK1) in the primary tumor has been found to be associated with reduced survival times and increased lymph node metastasis in breast cancer patients.

**Methods:**

In order to survey possible tumorigenic properties of GIRK1 overexpression, a range of malignant mammary epithelial cells, based on the MCF-7 cell line that permanently overexpress different splice variants of the *KCNJ3* gene (GIRK1a, GIRK1c, GIRK1d and as a control, eYFP) were produced. Subsequently, selected cardinal neoplasia associated cellular parameters were assessed and compared.

**Results:**

Adhesion to fibronectin coated surface as well as cell proliferation remained unaffected. Other vital parameters intimately linked to malignancy, i.e. wound healing, chemoinvasion, cellular velocities / motilities and angiogenesis were massively affected by GIRK1 overexpression. Overexpression of different GIRK1 splice variants exerted differential actions. While GIRK1a and GIRK1c overexpression reinforced the affected parameters towards malignancy, overexpression of GIRK1d resulted in the opposite. Single channel recording using the patch clamp technique revealed functional GIRK channels in the plasma membrane of MCF-7 cells albeit at very low frequency.

**Discussion:**

We conclude that GIRK1d acts as a dominant negative constituent of functional GIRK complexes present in the plasma membrane of MCF-7 cells, while overexpression of GIRK1a and GIRK1c augmented their activity. The core component responsible for the cancerogenic action of GIRK1 is apparently presented by a segment comprising aminoacids 235–402, that is present exclusively in GIRK1a and GIRK1c, but not GIRK1d (positions according to GIRK1a primary structure).

**Conclusions:**

The current study provides insight into the cellular and molecular consequences of *KCNJ3* overexpression in breast cancer cells and the mechanism upon clinical outcome in patients suffering from breast cancer.

**Electronic supplementary material:**

The online version of this article (doi:10.1186/s12885-016-2664-8) contains supplementary material, which is available to authorized users.

## Background

Four gene loci within the human genome encode for subunits of G-protein activated K^+^ channels (GIRK1-4). GIRK subunits form homo- or hetero-tetrameric ion channel complexes in the plasma membrane, act as classical G-protein effectors thereby mediating the regulation of cellular activity and/or excitability via hormones and neurotransmitters [1]. So far, known physiological roles of GIRKs comprise the vegetative regulation of the heartbeat, pain perception, learning and memory, anxiety behaviour and reward mechanisms [1]. Also in electrically non-excitable tissues physiological functions, like pancreatic insulin secretion [2, 3], blood platelet aggregation [4, 5] and regulation of lipid metabolism in fat cells [6] have been reported. Two of the gene loci encoding GIRK subunits in humans have been proven to be related to tumorigenesis and tumor growth: somatic mutations in the *KCNJ9* gene (encoding the GIRK4 subunit) have been identified to induce endocrine renal adenomas that cause primary aldosteronism and severe hypertension [7]. Overexpression of mRNA encoding the GIRK1 subunit, the product of the *KCNJ3* gene, may contribute significantly to the malignant properties of breast cancers: using expression profiling, Stringer et al. [8] observed that RNA derived from *KCNJ3* was aberrantly and highly overrepresented in primary invasive breast carcinomas when compared to the corresponding healthy breast tissue. GIRK1 mRNA overexpression correlated both with occurrence and number of lymph node metastases. Later on, Brevet et al. [9] observed a positive correlation between the immunohistochemical staining of GIRK1 in breast tumor specimen and lymph node metastasis and an inverse correlation with overall survival of the patients. A retrospective study, based on data from 905 invasive breast cancers derived from The Cancer Genome Atlas (TCGA) confirmed the findings delineated above at an appreciably larger scale. This corroborates the correlation between *KCNJ3* expression and breast cancer progression [10]. Malignant breast cancer cell lines express mRNAs encoding GIRK1 (but also GIRK2 and GIRK4) subunits [11] and several splice variants of the *KCNJ3* gene transcript [12]. In addition, the occurrence of GIRK1 and GIRK4 protein has been demonstrated in several breast cancer cell lines, including MCF-7 [12, 13].

Increasing evidence for *KCNJ3* expression in cancerous, compared to normal breast tissue and for its correlation with disease progression has accumulated. Comparatively little is known on a possible causal relationship between *KCNJ3* expression, tumorigenesis and cancer progression. GIRK1 protein may drive benign mammary epithelial cells (MECs) towards hallmarks of malignancy. In order to investigate a presumable role of GIRK1 in oncogenesis and metastasis of MECs, we overexpressed full length human GIRK1a as well as two splice variants, GIRK1c and GIRK1d (known to be abundant in breast cancer cells [12]), in the MCF-7 breast cancer cell line. This cell line was chosen, as GIRK1 mRNA levels are high, but expression of the corresponding protein(s) is low [12, 13] with the prospect to further strengthen potential malignant predicates due to pronounced overexpression. Analysis and comparison of selected vital parameters were performed in order to pinpoint characteristic features of MCF-7 that were possibly influenced by *KCNJ3* overexpression. By identification of peculiar properties that may be affected, we anticipated insight into the mechanism(s) how GIRK1 accomplishes its malignant task.

## Methods

### Solutions (concentrations in mmole/L): Zeroing Bathing Solution (ZBS)

K^+^/Asp^-^(120), KCl (20), MgCl_2_ (4), NaCl (10), EGTA^−^/K^+^ (10), HEPES^−^ (10), buffered with K^+^ to pH:7.4. *Pipette Filling Solution (PFS):* KCl (153), MgCl_2_ (4), CaCl_2_ (1), GdCl_3_ (0.2), HEPES^−^ (10) buffered with K^+^ to pH: 7.4. *Neutral buffered formalin (NBF):* 10 % formalin, PO_4_^−^ (75) buffered with Na^+^ to pH:7.0.

### Cell culture

MCF-7 cell line was obtained from ATCC (American Type Culture Collection) and maintained in minimal essential medium (MEM; Gibco, Life Technologies, Grand Island, NY, USA; Ordering No: 31095_029) supplemented with 10 % fetal bovine serum (Sigma Aldrich, St. Louis, USA, cat.No.: F2442), 1 mmole/L sodium pyruvate (Sigma Aldrich; St. Louis, USA, cat.No.: S8636) and penicillin/streptomycin (100 U.mL^−1^/100 ng.mL^−1^; Sigma Aldrich; St. Louis, USA, cat.No.: P0781) in 5 % CO_2_ atmosphere at 37 °C.

### Constructs

N-terminal (N-T) fusions of GIRK1a, GIRK1d and GIRK4 with enhanced yellow fluorescence protein (eYFP) and enhanced cyan fluorescence protein (eCFP) were expressed in MCF-7 cells using the pEYFP-C1 and pECFP-C1 based constructs described in detail in [12]. C-terminal (C-T) fusions of GIRK1a and GIRK1c with eYFP were produced by cloning the corresponding coding DNA sequence (CDS) into the plasmid pEYFP-N1 (Clontech Laboratories, Inc., Mountain View, CA, USA) using XhoI and EcoRI restriction sites. For fluorescence labelling of subcellular compartments plasmids encoding glycosylphosphatidyinositol/eCFP (GPI^eCFP^; for lipid rafts within plasma membrane [14]) and signal recognition particle receptor ß-subunit/eCFP (Srß^eCFP^; for endoplasmic reticulum (ER) [15]) were used. A vector for mammalian overexpression of fluorescence labelled G-protein β/γ subunits was generated by cloning Gγ_2_ CDS (Genbank Acc.No.: M37183) into the multiple cloning site (MCS) B of the pIRES vector (Clontech Laboratories, Inc., Mountain View, CA, USA) via XbaI and SalI restriction sites. Subsequently, the CDS of a fusion protein of eYFP with Gβ_1_ (Genbank Acc.No.: M313236; N-terminal with respect to Gβ_1_) was inserted into MSC B via NheI and EcoRI restriction sites. Integrity of the construct was verified by sequencing. Biological activity of fluorescence labelled G-protein β/γ subunits was verified by coexpression of the corresponding synthetic mRNAs in *Xenopus laevis* oocytes and subsequent electrophysiological testing for their ability to activate coexpressed GIRK ion channels composed of the GIRK1a and GIRK4 subunits (data not shown).

### Transfection

MCF-7 cells were transfected with the different constructs using TransFast reagent (Promega, Madison, USA, Cat. No.: E2341) and studied approx. 24 h after transfection. For stable transfection, pEYFP-C1 and pEYFP-N1 based constructs were linearized with AseI and pIRES construct with SalI, respectively, prior to transfection. Selection was started by adding 3 mg/mL G418 (Gibco, life technologies, Grand Island, USA, Ordering No.: 11811031) to the medium 24 h after the transfection. Single cell sorting was done two weeks after G418 addition. Individual clones were chosen by visual inspection using confocal Laser Scan Microscopy (cLSM). See Additional file 1: Table S1 for list of clonal cell lines that were used for the present study.

### Confocal laser scan microscopy

Fluorescence images of transfected MCF-7 cells were obtained in-vivo using Leica inverted microscope with 63x H_2_O immersion objective (NA: 1.20) with attached laser-scan module (DMIRE2 and TCS SL2; Leica Microsystems, Heidelberg, Germany) as described previously [12].

### qPCR

RNA isolation and cDNA synthesis were performed as described previously [12]. qPCR has been described in [16]. Primer sequences were as follows: *GIRK1a_f:* 5′-GTGGAAACAACTGGGATGAC-3′; *GIRK1a_r:* 5′-GTTGCATGGAACTGGGAGTA-3′; *GIRK1c_f:* 5′- CAAGCTGCTCAAATCTCGGC-3′; *GIRK1c_r:* 5′-AGTTGATCTGCCCCTGTACT-3′; *GIRK1d_f:* 5′-CAAGCTGCTCAAAGGATGAC-3′; *GIRK1d_r:* 5′-GTTGCATGGAACTGGGAGTA-3′; *GAPDH_f:* 5′-ATGGGGAAGGTGAAGGTCG-3′; *GAPDH_r:* 5′-GGGGTCATTGATGGCAACAATA-3′.

### Immunohistochemistry (IHC)

Cells were fixed in NBF, embedded in agarose gel (7 %) and then processed for paraffin embedding. Target retrieval solution (pH: 9.0; Dako, Glostrup, Denmark; Product No: S236884) heated for 40 min at 150 W in a microwave was used for antigen retrieval. Slides were then washed in washing buffer (Dako, Glostrup, Denmark; Product No: S3006) and incubated with a monoclonal antibody against GIRK1 (Abcam, Cambridge, UK; cat.No: 119246; 1:50; clone 3E11). For visualization, the EnVision + dual link reagent (rabbit/mouse horse radish peroxidase, Glostrup, Dako, Denmark; Product No: K406311) was used according to manufacturer’s protocols. Immunohistochemical staining was developed by incubation of sections with diaminobenzidine (DAB; Glostrup, Dako; Product No: K406511) as a chromogenic substrate. Slides were then washed in Dako wash buffer, counterstained with Meyer’s hematoxylin (from the pharmacy of the Medical University of Graz), rinsed in tap water, dehydrated and mounted with Entellan® (Merck, Darmstadt, Germany). Sections incubated without primary antibody served as negative controls.

### Analysis of vital parameters of cell lines

In order to avoid possible deviations of vital parameters that might be due to the cloning procedure itself instead of differential overexpression of GIRK1 variants, assessment of these parameters was always conducted on more than one cell line overexpressing identical constructs (Additional file 1: Table S1). As no difference in vital parameters between cell lines expressing identical GIRK1 variants was observed, these data were pooled and analyzed collectively. In order to monitor eventual effects of stable eYFP overexpression alone or of the manipulation of cellular genome on the vital parameters tested, all vital assays were performed using both MCF-7^WT^ and MCF-7^eYFP^ as controls.

### Adhesion assay

MCF-7 cells were washed with PBS and plated into each well of Corning® BioCoat™ Fibronectin 96 Well Clear Flat Bottom (Corning, NY. USA, Cat No: 354409). Non-adherent cells were removed 150 min later by washing with PBS. Adherent cells were fixed with 2 % formaldehyde, air dried and stained with 0.1 % crystal violet (Sigma Aldrich, St.Louis, USA; Cat No: C0775) in PBS. Bound dye was solubilized with 10 % acetic acid and absorbance was measured at 550 nm using a plate reader (Labsystem Mutiskam MS). Cell-free wells served as blanks.

### Proliferation assay

Cells were plated in six well plates and incubated in a cell culture incubator until reaching 80 % confluency. Proliferation of the cells were assessed based on the incorporation of the thymidine analog, 5-bromo-2′-deoxyuridine (BrdU) into the newly synthesized DNA of replicating cells (during S phase). Labeling of DNA was done by adding 10 μL of BrdU solution directly to each mL of cell culture media. (APC BrdU flow kit; BD Pharmingen, San Diego, CA, USA; Cat No: 552598). The treated MCF-7 cells were then incubated for 3 h in cell culture incubator. The cells were then stained with Anti-BrdU APC. 7-AAD (7-Aminoactinomycin D) (DNA binding dye) used in order to define cell cycle (G_1_/G_0_, S, G_2_/M).

### Invasion assay

Corning® BioCoat™ Matrigel® Invasion Chambers (Corning, NY. USA, Cat No: 354480) were rehydrated in MEM for 2 h at 37 °C. 1.25x10^5^ cells/mL in 2 mL MEM (without fetal bovine serum (FBS)) were seeded into the upper compartment and 2 mL MEM with 5 % FBS as chemoattractant were added to the lower compartment of Matrigel. After 24 h incubation in cell culture incubator, non-invading cells were removed from the upper surface of the membrane by scrubbing with cotton tipped swabs. To stain the invading cells, membranes beneath the insert were cut, fixed with ice-cold methanol and stained with 0.1 % crystal violet. Invading cells were counted under microscope.

### Wound healing assay

Cells were plated into 24 well plates (1x10^5^ cells/well) and incubated for 24–72 h to achieve a confluent monolayer. The cell monolayer was scratched in a straight line with a pipet tip (VWR, USA; Cat No #53508-910). Debris was removed by washing the cells once with PBS followed by adding MEM growth medium. The plate was put into the cell observer (Zeiss Axiovert 200 M). Images were taken over the course of 72 h at 1 h interval. ImageJ software was used for analysis of the resulting time lapse videos [17].

### Motility assay

Cellular velocities and motility coefficients were assessed by cell observer (Axiovert200M, Zeiss, Germany) over a time period of 72 h, as described previously [16].

### Ex ovo chorioallantoic membrane (CAM) assay

Fertilized white leghorn chicken eggs from local hatchery (Schropper GmbH, Gloggnitz, Austria) were incubated at 37.6 °C and 70 % humidity (J. Hemel Brutgeräte GmbH & Co KG, Am Buschbach, Germany). The egg shell was cracked on day 3 of chicken embryo development, the embryo was decanted to a sterile dish and further incubated as indicated before. On day 10 of cultivation cell onplants with volumes of 20 μl were applied on vascular branches of the CAM within sterile silicon rings of 5 mm diameter (1x10^6^ cells in 10 μl PBS mixed 1:2 with Matrigel® (Corning, NY, USA, cat.No: 356237), allowing subsequent tumor growth for 3 days [18]. The intensity of the angiogenic response was analyzed under a stereomicroscope according as described previously [19].

### Electrophysiology

Single channel recording from MCF-7 cells in the cell attached configuration was performed exactly as described previously for PIEZO1 mechanosensitive ion channel protein, but without application of mechanical stress to the membrane [16].

### Statistics

Statistical evaluation was performed using SPSS software in the SigmaStat environment (SigmaPlot 13.0; Systat Software GmbH, Erkrath, Germany) or using “R” software (version 3.2.1; https://www.r-project.org/). Depending on variance and distribution of the dataset, the appropriate tests were performed, as specified in the legends to the figures.

## Results

### Characterization of the generated MCF-7 based cell lines

**Fig. 1:**
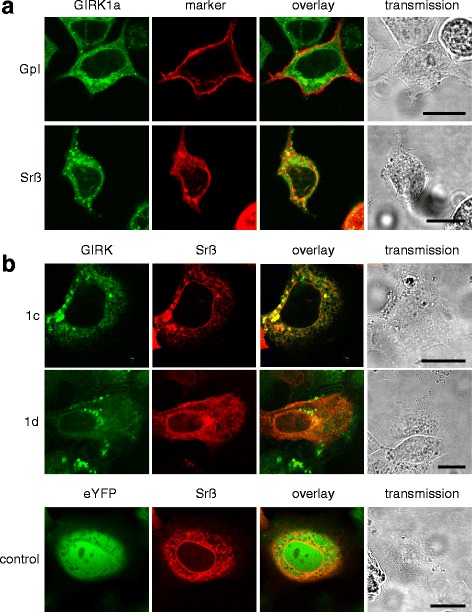
cLSM in-vivo reveals overexpressed GIRK1 protein to localize in a large part to the ER. Horizontal sequences of images show identical cells. The sequence of channels (from left to right is: eYFP (*green*)/ eCFP (*red*)/ overlay / transmission. Scalebar : 15 μm in all images. **a** *Upper sequence*: subcellular localization of GIRK1a, labelled with eYFP at the C-terminus (MCF-7^GIRK1a^; transient transfection with GpI-eCFP was used as marker for lipid rafts in the plasma membrane). *Lower sequence*: subcellular localization of GIRK1a, labelled with eYFP at the C-terminus (MCF-7^GIRK1a^; transient transfection with Srß-eCFP was used as ER marker). **b** *Upper sequence*: subcellular localization of hGIRK1c, labelled with eYFP at the C-terminus (MCF-7^GIRK1c^; transient transfection with Srß-eCFP was used as ER marker). *Middle sequence*: subcellular localization of hGIRK1d, labelled with eYFP at the N-terminus (MCF-7^GIRK1d^; transient transfection with Srß-eCFP was used as ER marker). *Lower sequence*: subcellular localization of eYFP alone (MCF-7^eYFP^; transient transfection with Srß-eCFP was used as ER marker)

**Fig. 2:**
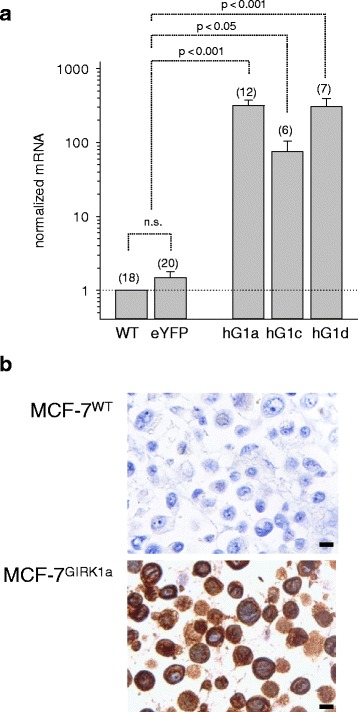
Overexpression of GIRK1 mRNA and protein in the stably transfected MCF-7 cells assessed by IHC. **a** Quantification of mRNA expression encoding different GIRK1 variants via qPCR in stably transfected cells. Expression was normalized to the expression level in the MCF-7^WT^ cell line. *WT*: MCF-7^WT^; *eYFP*: data derived from two cell lines with stably integrated pEYFPN1 vector alone (MCF-7^eYFP^); *hG1a*: data derived from two cell lines overexpressing N-terminal and two cell lines overexpressing C-terminal fusions of eYFP with the GIRK1a protein (MCF-7^GIRK1a^); *hG1c*: data derived from two cell lines overexpressing C-terminal fusions of eYFP with the GIRK1c protein (MCF-7^GIRK1c^); *hG1d*: data derived from two cell lines overexpressing N-terminal fusions of eYFP with the GIRK1d protein (MCF-7^GIRK1d^). Mean values ± SEM were plotted (number of experiments is given in parenthesis above each bar). Statistical significances between groups are indicated. hG1a, as well as hG1d differs from both WT, as well as from eYFP, statistically significant at the *p* < 0.001 level. hG1c differs from both WT, as well as from eYFP, statistically significant at the *p* < 0.05 level. Kruskal-Wallis one way analysis on ranks was used for analysis of statistical significance. **b** Detection of GIRK1a protein in stably transfected cells via immunohistochemistry. *Brown*: Immunoreactivity. *Blue*: hematoxylin stain. Scale bar corresponds to 100 μm throughout. *Upper image*: MCF-7^WT^; *Lower image*: MCF-7^hGIRK1a^

Effective overexpression of GIRK1 protein in the stably transfected MCF-7 cell lines was verified by several independent methods. GIRK1 is an integral membrane protein with two transmembrane α-helices per subunit; therefore, the subcellular localization of fluorescent chimaeras in membranes is expected to indicate successful overexpression of the entire protein. Indeed, expression of C-terminal labelled GIRK1a was different compared to soluble eYFP alone, GIRK1 being located mostly in the endoplasmic reticulum (ER) and, to some extent, also in the plasma membrane, but not in the nucleus. Pure eYFP distributed evenly within the cytosol and the nucleus, with the exception of vacuoles (Fig. 1). Localization identical to C-terminal labelled GIRK1a was also observed for N-terminal labelled one, indicating that the position of the fluorophore did not affect the proteins’ synthesis or subcellular localization (Additional file 1: Figure S1). Similar subcellular localization was observed for the shorter splice variants GIRK1c and GIRK1d (Fig. 1). In addition to fluorescence microscopy, qPCR corroborated overexpression of mRNAs encoding all GIRK1 variants in the established cell lines (Fig. 2a; Additional file 1: Figure S2). Since the epitope recognized by the antibody used is present exclusively in full length GIRK1a, IHC may not be considered a suitable tool to confirm expression of the GIRK1c/1d as these splice variants lack the corresponding part of the C-terminal portion, respectively. Under our experimental conditions native MCF-7^WT^ cells did not exert detectable GIRK1a expression in IHC, underscoring the scale of protein overexpression in MCF-7^GIRK1a^ cells by orders of magnitude; these results are in line with qPCR results (Fig. 2a). Finally, IHC clearly demonstrated overexpression of the protein as well as its subcellular localization (Fig. 2b). Thereupon we conclude that the cell lines generated represent valid models to study the biological effect(s) of GIRK1 variant overexpression in human breast cancer.

### Effects of GIRK1 overexpression on adhesion and proliferation of MCF-7 cells

**Fig. 3:**
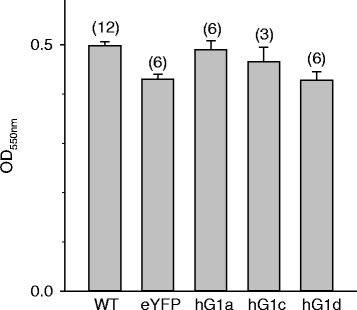
Surface adhesion of MCF-7 cells is unaffected by GIRK1 overexpression. Quantification of cells adhering to fibronectin coated substrate via OD_550nm_. *WT*: MCF-7^WT^; *eYFP*: MCF-7^eYFP^; *hG1a*: MCF-7^GIRK1a^; *hG1c*: MCF-7^GIRK1c^; *hG1d*: MCF-7^GIRK1d^. Mean values ± SEM were plotted (number of experiments is given in parenthesis above each bar). The mean values do not differ statistically significantly

**Fig. 4:**
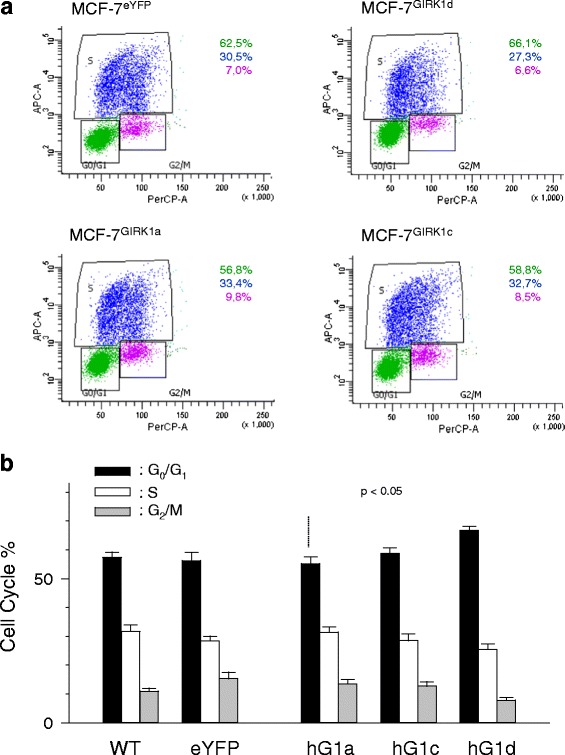
Survey of cell cycle and proliferation upon GIRK1 overexpression in MCF-7 cells. **a** Original results from the assessment of cell cyle using gated cell sorting according to fluorescence intensities for PerCP-A (*x*-*axis*) and APC-A (*y*-*axis*) for different experimental groups. % of cells for the given experiment is stated in respective colors besides the plot. **b** *Statistics for the percentage of time spent in the different phases of the cell cycle* Mean values ± SEM were plotted. N was (in parenthesis behind each experimental group): was: MCF-7^WT^ (8) / MCF-7^eYFP^ (12) / MCF-7^GIRK1a^ (16) / MCF-7^GIRK1d^ (12) / MCF-7^GIRK1d^ (6). G_1_/G_0_ fraction of MCF-7^GIRK1d^ differs statistically significant at the *p* < 0.05 level from the one of MCF-7^GIRK1a^. One way ANOVA was used for analysis of statistical significance

Modification of selected cellular vital parameters in culture by candidate proteins indicates whether a protein under consideration may act as an oncoprotein causing and promoting particular features of malignancy [20, 21]. Cell adhesion (Fig. 3) remained unaffected upon overexpression of GIRK1a, GIRK1c and GIRK1d variants. When monitoring cell cycle and proliferation via gated cell sorting, it became evident that the parameters tested where, for the major part, unaffected (Fig. 4). The exception was MCF-7^GIRK1d^, that exerted an increased period between cell division and start of DNA replication (G_0_/G1) when compared to all other groups. This increase was moderate and statistically significant only by comparison with MCF-7^GIRK1a^. The difference is in line with our observation (SR; AG) that MCF-7^GIRK1d^ cells require longer time intervals to grow, when compared to the other cell lines under identical conditions. We conclude that upon GIRK1 overexpression, proliferative signaling remained practically unchanged in MCF-7 under our experimental conditions.

### GIRK1 overexpression interferes with wound healing and invasion

**Fig. 5:**
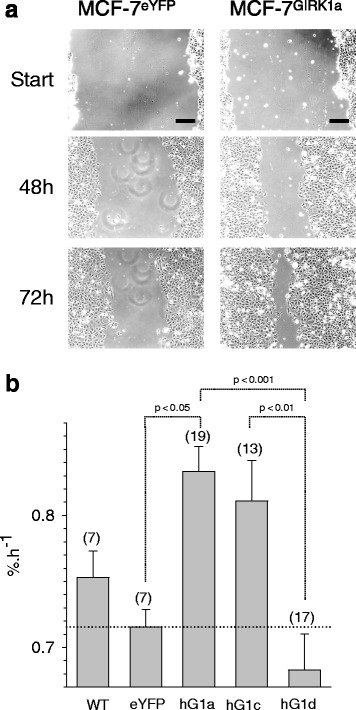
GIRK1 overexpression affects wound healing rate in a differential manner, depending on the variant tested. **a** Representative view of monolayers at different time intervals after scratching. Scale bars correspond to 200 μm. *Left*: control; *right*: GIRK1a overexpressors. **b** Wound healing rates (in % healing per h). *WT*: MCF-7^WT^; *eYFP*: MCF-7^eYFP^; *hG1a*: MCF-7^GIRK1a^; *hG1c*: MCF-7^GIRK1c^; *hG1d*: MCF-7^GIRK1d^. Mean values ± SEM were plotted (number of experiments is given in parenthesis above each bar). Statistical significances between groups are indicated. hG1a differs from eYFP statistically significant at the *p* < 0.05 level. hG1a differs from hG1d statistically significant at the *p* < 0.001 level. hG1c differs from hG1d statistically significant at the *p* < 0.01 level. Kruskal-Wallis one way analysis on ranks was used for analysis of statistical significance

**Fig. 6:**
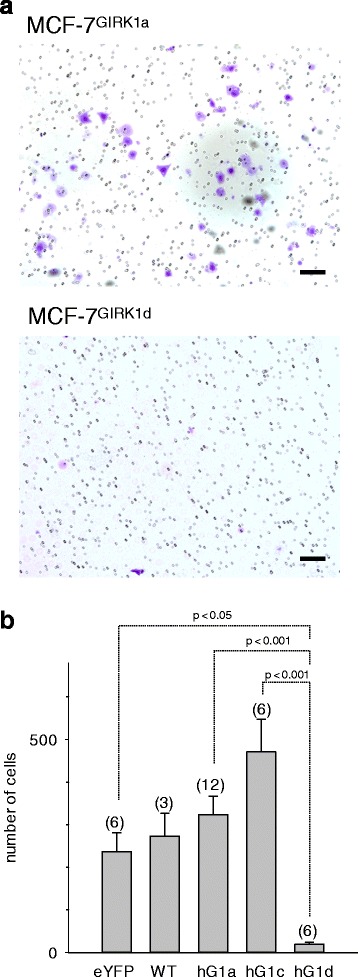
Effects of GIRK1 variant overexpression on chemoinvasion through Matrigel. **a** Comprehensive view of fixated cells, stained with crystal violet, that managed to invade the Matrigel layer (10x magnification). The Scalebar corresponds to 100 μm for both images. *Upper*: MCF-7^GIRK1a^, *lower*: MCF-7^GIRK1d^. **b** Quantification of the number of invasive cells after 24 h. *WT*: MCF-7^WT^; *eYFP*: MCF-7^eYFP^; *hG1a*: MCF-7^GIRK1a^; *hG1c*: MCF-7^GIRK1c^; *hG1d*: MCF-7^GIRK1d^. Mean values ± SEM were plotted (number of experiments is given in parenthesis above each bar). Statistical significant differences between groups are indicated. hG1d differs from eYFP statistically significant at the *p* < 0.05 level. hG1a and hG1c differ from hG1d statistically significant at the *p* < 0.001 level. Kruskal-Wallis one way analysis on ranks was used for analysis of statistical significance

Both wound healing and tumor development, two processes that may seem unrelated at the first glance, are based on similar molecular mechanisms and signaling pathways [22]. Wound healing, however, is a rather transient process, while tumors pursue to evolve and spread. Hence, wound healing assay was performed to identify additional characteristic vital parameters, potentially affected by GIRK1 expression. As shown in Fig. 5a the rate of wound closure was markedly enhanced by overexpression of the full length GIRK1a protein when compared to control (see also Additional files 2, 3 and 4). While overexpression of GIRK1c produced a similar, albeit statistically not significant increase, overexpression of GIRK1d did not result in an increase of wound closure rate that was even slightly reduced when compared to control (Fig. 5b). Next, the Matrigel invasion assay regarded to be indicative for activation of invasion and metastasis was performed. This assay unveiled that GIRK1 overexpression affected invasion towards a chemoattractant in a bimodal manner, depending on the respective splice variant: overexpression of GIRK1d greatly reduced the number of cells with invasive phenotype, while overexpression of GIRK1a and GIRK1c slightly promoted invasion, although not statistically significant (Fig. 6; see Additional file 1: Figure S3 for representative micrographs of all the groups tested). Taken together, both assays uncover remarkable differences between the larger, higher molecular mass, splice variants GIRK1a and GIRK1c, which significantly promoted wound healing and invasive phenotype when compared to GIRK1d.

### Cellular motilities and velocities are affected by GIRK1 overexpression

**Fig. 7:**
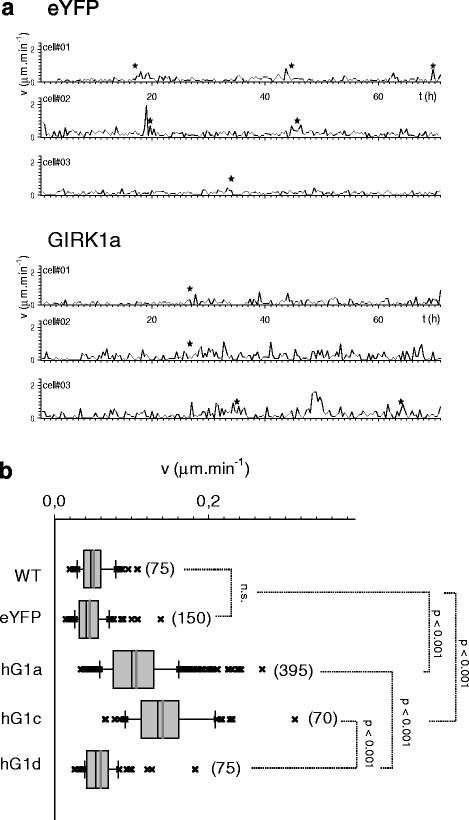
Effect of GIRK1 variant overexpression on single cell velocities. **a** Cellular velocities for three representative cells during the entire observation interval of 72 h. Asterisks denote cell divisions. *Upper*: MCF-7^eYFP^; *lower*: MCF-7^GIRK1a^. **b** Graphical representation of mean cellular velocities for different experimental groups. *WT*: MCF-7^WT^; *eYFP*: MCF-7^eYFP^; *hG1a*: MCF-7^GIRK1a^; *hG1c*: MCF-7^GIRK1c^; *hG1d*: MCF-7^GIRK1d^. The median value is represented by the black line within the box, box margins represent 75 and 25 % percentiles, whiskers indicate 90 and 10 % percentiles, values outside the 10–90 % interval are plotted individually as crosses. The grey line represents the mean value. The number of individual cells is given in parenthesis besides each box. Statistical significant differences between groups are indicated. hG1a, as well as hG1c, differ from both WT, as well as from eYFP, statistically significant at the *p* < 0.001 level. hG1d differs from both hG1a, as well as from hG1c, statistically significant at the *p* < 0.001 level. Kruskal-Wallis one way analysis on ranks was used for analysis of statistical significance

**Fig. 8:**
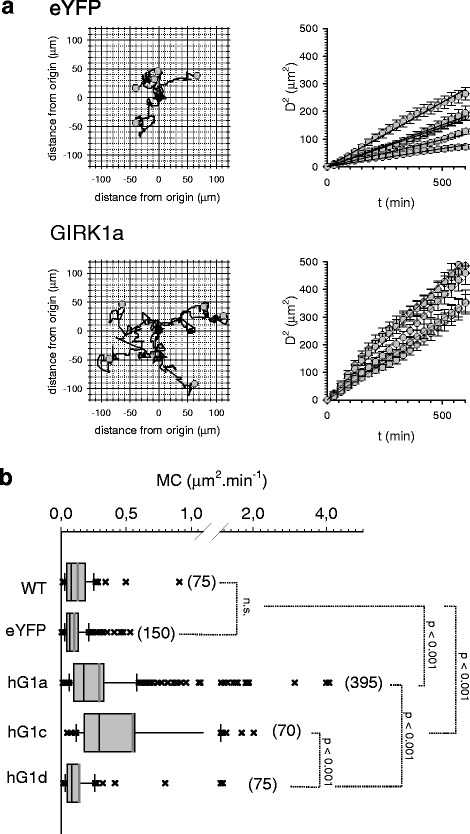
Effect of GIRK1 variant overexpression on single cell motility coefficients. **a** *Left panel*: Flower plots showing the trajectories of 5 representative cells for the entire observation interval of 72 h (*black* line). Starting position of each individual cell was set to zero coordinates (*black* circle). *Grey* circles indicate the positon of an individual cell after 72 h. *Right panel*: Squared distance as a function of time for the five cells shown to the left (circles; bars indicate standard error). Solid lines represent a linear fit through the data, resulting in the motility coefficient (MC). *Upper*: MCF-7^eYFP^; *lower*: MCF-7^GIRK1a^. **b** Graphical representation of cellular motility coefficients for different experimental groups. *WT*: MCF-7^WT^; *eYFP*: MCF-7^eYFP^; *hG1a*: MCF-7^GIRK1a^; *hG1c*: MCF-7^GIRK1c^; *hG1d*: MCF-7^GIRK1d^. The median value is represented by the black line within the box, box margins represent 75 and 25 % percentiles, whiskers indicate 90 and 10 % percentiles, values outside the 10–90 % interval are plotted individually as crosses. The grey line represents the mean value. The number of individual cells is given in parenthesis besides each box. Statistical significant differences between groups are indicated. hG1a, as well as hG1c, differs from both WT, as well as from eYFP, statistically significant at the *p* < 0.001 level. hG1d differs from both hG1a, as well as from hG1c, statistically significant at the *p* < 0.001 level. Kruskal-Wallis one way analysis on ranks was used for analysis of statistical significance

Both wound healing and Matrigel invasion assays gather properties of cells related to cell migration in vitro, proliferation, cell/extracellular-matrix and cell/cell interactions [23, 24]. When cellular velocities were directly quantified it became evident that average cellular velocities were greatly augmented upon overexpression of GIRK1a and GIRK1c, when compared to control (MCF-7^eYFP^), MCF-7^WT^ and MCF-7^GIRK1d^ (Fig. 7). Average velocities of MCF-7^GIRK1d^ cells were indistinguishable from MCF-7^WT^ or control cells. Similar results were obtained for cellular migration, as depicted by cellular motility coefficients (MCs) that were also considerably increased by GIRK1a and GIRK1c overexpression (see Additional files 5 and 6). Again, cellular MCs were indistinguishable when MCF-7^GIRK1d^, MCF-7^WT^ and control cells were compared (Fig. 8). In order to rule out the remote possibility that differences in average cellular velocities and MCs observed were produced by differences between individual MCF-7 cell clones, MCF-7^WT^ cells were transiently transfected with GIRK1a and the corresponding control plasmid. Time-lapse microscopy revealed that GIRK1a transfected cells exhibited massively elevated average velocities and MCs, when compared to control cells (transfected with eYFP alone) or non-transfected ones (Additional file 1: Figure S4).

### GIRK1 overexpression affects angiogenesis

**Fig. 9:**
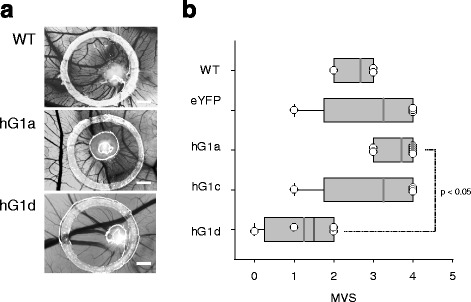
GIRK1d overexpression reduces angiogenesis in the CAM assay. **a** Representative view of cellular onplants and vascularization. The silicone ring, used to initially stabilize the onplant is also visible. Scale bars correspond to 200 μm for all images. **b** Macroscopic vascularization scores (MVS) for the different experimental groups. *WT*: MCF-7^WT^; *eYFP*: MCF-7^eYFP^; *hG1a*: MCF-7^GIRK1a^; *hG1c*: MCF-7^GIRK1c^; *hG1d*: MCF-7^GIRK1d^. The median value is represented by the black line within the box, box margins represent 75 and 25 % percentiles, whiskers indicate 90 and 10 % percentiles, values outside the 10–90 % interval are plotted individually as crosses. The *grey* line represents the mean value. The statistically significant difference between MCF-7^GIRK1d^ and MCF-7^GIRK1a^ is indicated (*p* < 0.05). Kruskal-Wallis one way analysis on ranks was used for analysis of statistical significance

Induction of angiogenesis represents another peculiar feature of cancer cells: while absent in normal tissue, except for embryogenesis, wound healing and endometrial cycling, the “angiogenic switch” is permanently turned on in tumor cells in order to provide tumor associated neovasculature [21]. To study the potential of the different MCF-7 based cell lines to induce angiogenesis, the CAM assay was performed (Fig. 9). No apparent difference in macroscopic vascularization scores (MVSs) was found for GIRK1 overexpressors when compared individually to either control (MCF-7^eYFP^) or MCF-7^WT^. MVS was, however, substantially decreased in MCF-7^GIRK1d^ cells when they are compared to MCF-7^GIRK1a^ that showed the highest MVSs amongst all the cell lines studied. This finding suggests that the “angiogenic switch” becomes down-regulated by GIRK1d overexpression. Alternatively, GIRK1a overexpression has no effect or may even increase angiogenesis, when compared to MCF-7^WT^ cells that have moderate angiogenic activity per se (see Fig. 9a and as an example, reference [25]).

### Are there functional GIRK ion channels in MCF-7 cells?

In order to investigate whether GIRK1 is able to form functional K^+^ ion channels in the plasma membrane of MCF-7 cells, single channel recording was performed. At present limited information is available on G-protein coupled receptors (GPCRs) that may activate GIRKs in the MCF-7 cell line. Therefore we produced a MCF-7 based cell line, permanently overexpressing free G-Protein β/γ subunits (Gβ/γ). Free Gβ/γ is known to activate GIRK channels even in the absence of GPCR activation (MCF-7^Gβγ^; Additional file 1: Figure S5) [1]. Gβ/γ induced K^+^ channel activity could be observed only in one single patch out of 53 experiments, making further experimentation unpromising. Transient overexpression of GIRK1 and GIRK4 subunits increased the frequency of observations of this K^+^ channel activity in MCF-7^Gβγ^ to some extent, betokening that it had been produced by GIRK protein expression (Additional file 1: Figure S5). Of note a K^+^ channel with comparable properties has not yet been observed in MCF-7^WT^ cells (Table 1). This observation suggests that endogenous GIRKs do not display appreciable activity in the absence of GPCR activation. In summary we have demonstrated that functional GIRK ion channels can be formed in MCF-7 cells, nevertheless their abundancy is, in the absence of ancillary overexpression, apparently very low.

**Table 1:** Frequency of observations of functional GIRK channels in the cell lines used

	N patches	N with GIRKs	% with GIRKs
MCF-7^WT;a^	291	0	0 %
MCF-7^Gβγ^	53	1	2 %
MCF-7^Gβγ^*hG1a *hG4	10	2	20 %

^a^:data from reference [16]

## Discussion

Our results clearly corroborate that overexpression of GIRK1 protein exerts profound effects on wound healing, chemoinvasion and cellular motility in the MCF-7 breast cancer cell line suggesting a role to promote invasion and metastasis. Induction of angiogenesis was also affected. Most noteworthy is the fact that all vital parameters affected by GIRK1 overexpression are manipulated in opposite direction, depending on the GIRK1 variant tested. Overexpression of either GIRK1a or GIRK1c reinforces vital properties of MCF-7 cells towards the malignant phenotype, while GIRK1d overexpression seemingly counteracts upon the opposite direction. Hence, differential features of GIRK1 variant proteins could be responsible for this antithetical behavior and comparison of their established functional properties may provide insight. While homo- and heterotetrameric K^+^ channels containing the full length GIRK1a subunit have lengthily been studied [1], little is known on the function(s) and essentially nothing on the possible (patho)physiological role of the smaller GIRK1c and GIRK1d variants. In the few studies undertaken so far by several groups using different expression systems, homotetramers composed of GIRK1c or GIRK1d subunits proved themselves to be inactive as ion channels (despite of expression at the protein level), and, in addition, entirely silenced both homo- and heterotetrameric GIRK complexes by acting as dominant negative constituents (see [12] for functional testing of splice variants and a subsumption of existing literature). Thereupon we suggest that the effect of GIRK1d that is contrary to the effect of GIRK1a overexpression is due to the dominant negative effect of GIRK1d on the function of GIRK complexes. Reinforcement of the malignant phenotype via GIRK signaling takes, to some degree, already place in the native MCF-7^WT^ cell line, where both mRNA and protein have been shown to exist [12, 13], although at a much smaller scale when compared to the overexpressors. Overexpression of GIRK1d might impair endogenous GIRK signaling in MCF-7^WT^, weakening cellular behavior related to the activation of invasion, metastatic spread and induction of angiogenesis. At the same time, overexpression of GIRK1a would enhance and reinforce the biological activities of preexisting GIRK complexes, in line with the results of the prevailing study. Also the finding of prolonged G_0_/G_1_ period in MCF-7 cells upon GIRK1d overexpression supports the view of a dominant negative action on endogenous GIRK complexes. The functional role of GIRK1c overexpression remains, however, enigmatic. Our study reveals for the first time a function for the GIRK1c variant, other than the constitutive negative properties reported in previous publications. Here we report that the functional outcome of GIRK1c overexpression rather resembles the one produced by overexpression of the GIRK1a subunit. Indeed all variants of GIRK1 comprise the integral transmembrane part, including permeation pathway and ion selectivity filter required to catalyze K^+^ permeation across the plasma membrane. Hence, truncated splice variants of GIRK1 may, under our conditions, act as K^+^ channels, although this has previously never been observed. At present, biological activities, other than K^+^ permeation across the plasma membrane, might mediate the biological effects observed. By alternative splicing, full length mRNA encoding GIRK1a is composed of three different exons, i.e. exons 1–3 [26]. GIRK1c mRNA comprises exons 1 and 2, while the one encoding GIRK1d is assembled from exons 1 and 3 (exon 2 is missing) [12]. At the protein level, GIRK1a contains 501 amino acids. All three GIRK1 variants share amino acid positions 1–234 at the N-terminus. Due to a frameshift that prevents translation of exon 3, GIRK1d has one single additional C-terminal amino acid (glycine; position 235). In contrast and due to exon 2, GIRK1c shares amino acids positions 235–402 with GIRK1a. To sum up, the difference between GIRK1c and GIRK1d are 167 additional amino acids at the C-terminal of GIRK1c, when compared to the single additional amino acid 235 of GIRK1d. Thus, the key to the tumor promoting activity of GIRK1 must conceivably be located in the amino acid segment 235–402. It must be mentioned that the subcellular distribution observed, i.e. the major fraction of GIRK1 protein remaining within intracellular membranes rather than in the plasma membrane, is, at the first glance surprising. It is, however, identical to that reported previously upon *transient* transfection of MCF-7 cells with GIRK1 splice variants [12]. It has been frequently observed in studies dealing with GIRK1 synthesis, trafficking, and plasma membrane insertion that homooligomeric GIRK1 tetrameric protein remains mostly located in intracellular membranes whereas heteromeric assembly with other GIRK isoforms results in partial plasma membrane insertion and glycosylation of the GIRK1 subunit [27–29]. It was, however, observed that even in native cells and in the presence of additional GIRK isoforms as heterooligomerization partners [30–33] at least 64 % of GIRK1 protein remain permanently confined to intracellular membranes [32, 34]. Although the (patho)physiological role of intracellular GIRK1 repositories within the ER of malignant MECs described here remains obscured, their existence is in line with the one generally observed and we can, at present, not decide whether intracellular or plasma membrane located GIRK protein is responsible for the effects observed by us. Also worth mentioning at this point are long noncoding RNAs (lnRNAs), sometimes even mRNAs, that do not require protein to be synthesized at all and have been found to shift the phenotype of cancer cells towards malignancy [35]. In the current study, however, the overexpressed mRNAs were devoid of their 3′- and 5′-untranslated regions (UTRs) which presumably are crucial for such activities. Also the fact that IHC gives negative results for MCF-7^WT^ cells does not allow to rule out GIRK1 protein(s) as being responsible for the biological effects observed in wild-type and control MCF-7 cells and to favor the InRNA hypothesis. As signal transduction molecules such as GIRK complexes exert their biological activities usually at very low abundancies, immunoreactivity below detection threshold cannot be regarded as proof for the absence of protein. Therefore we favor the hypothesis that the tumor promoting effect of *KCNJ3* overexpression is provoked by the corresponding protein(s). Searching for a potential liaison between GIRK complexes in the plasma membrane, cancerogenesis and cancer progression, two major connections are obvious: First, K^+^ channel proteins have been found to promote pathophysiological phenotypes responsible for malignant growth of cancer cells in a vast amount of reports (see [36–39] for review). While some of these studies have identified K^+^ channels to enhance proliferation, others reported on reinforcement of angiogenesis and cellular motility, as described in the present study [20]. K^+^ permeation as well as other hitherto unknown functions of K^+^ channel proteins (called *“moonlighting”* functions) had been found to promote the malignant phenotype [40–43]. The second potential relation of GIRK signalling to cancer is exclusive amongst K^+^ channel proteins as GIRK complexes act as direct G-protein effectors. For example, GPCR/G-protein mediated signalling guides the migration of metastatic breast tumor cells towards bone tissue that, in turn, forms spatial and environmental niches promoting tumor grow in response to factors released by the invaders [44, 45]. In general terms, pathological GPCR signaling has long-since been identified as a major target in the development of novel therapeutic approaches [46, 47]. As shown in the prevailing study, GIRK complexes are able to function as K^+^ channels, but occur at extremely low abundancy in MCF-7 cells. Moreover, GIRK activation depends to a substantial extent on freely available G-protein β/γ dimers, i.e. GPCR activation. We conclude that the oncogenic potential of GIRK1 overexpression is closely linked to GPCR signaling. At present we cannot discriminate between an impact of K^+^ permeation itself or another, hitherto unknown biological function. Since also GIRK1c, the subunit that so far has not been observed to function as an ion channel, exerts biological activity similar to the one of GIRK1a, one may favor the latter hypothesis. We can, however, not rule out the possibility that the GIRK1c subunit is functional as an ion channel in MCF-7 cells. More experimentation is required to arrive to definite conclusions concerning this aspect.

## Conclusions

This is the first study to provide insight into the cellular and molecular consequences of *KCNJ3* overexpression in breast cancer cells and the mechanism how overexpression could translate into the worsened clinical outcome in breast cancer. Further research should be devoted to elucidate the molecular chain of events leading to reinforcement of malignant phenotype by *KCNJ3* overexpression observed in this study. Furthermore the investigation of the suitability of GIRK1 mRNA and∕or protein as clinical biomarker(s) as well as the usefulness of the GIRK1 protein as putative therapeutic target becomes worth striving for.

## Abbreviations

CAM, chorioallantoic membrane; C-T, carboxy terminus; eCFP, enhanced cyan fluorescence protein; ER, endoplasmic reticulum; eYFP, enhanced yellow fluorescence protein; FBS, fetal bovine serum; GIRK1, G-protein activated inwardly rectifying K^+^ channel, subunit 1; GIRK1a, GIRK1c and GIRK1d, Splice variants *a*, *c* and *d* of the G-protein activated inwardly rectifying K^+^ channel, subunit 1; GIRK4, G-protein activated inwardly rectifying K^+^ channel, subunit 4; GPCR, G-protein coupled receptor; GpI, glycosylphosphatidylinositol; Gβ1, G-protein β1 subunit; Gγ2, G-protein γ2 subunit; IHC, immunohistochemistry; KCNJ3, Gene encoding G-protein activated inwardly rectifying K^+^ channel, subunit 1; KCNJ9, Gene encoding G-protein activated inwardly rectifying K^+^ channel, subunit 4; MC, cellular motility coefficient; MCF-7, Michigan Cancer Foundation cell line 7; MCS, multiple cloning site; MEC, mammary epithelial cell; MEM, minimum essential medium; MVS, macroscopic vascularization score; N-T, amino terminus; qPCR, quantitative polymerase chain reaction; RNA, ribonucleic acid; Srß, signal recognition particle receptor ß-subunit

## Additional files

Additional file 1: Supplementary Figures. (PDF 1221 kb) Additional file 2: Wound healing (MCF-7^eYFP^). (AVI 11871 kb) Additional file 3 Wound healing (MCF-7^GIRK1a^). (AVI 13795 kb) Additional file 4: Wound healing (MCF-7^GIRK1d^). (AVI 13569 kb) Additional file 5: Velocity & Motility (MCF-7^eYFP^). (AVI 16420 kb) Additional file 6: Velocity & Motility (MCF-7^GIRK1a^). (AVI 23421 kb)
