# Supplementary material for: Overexpression of KCNJ3 gene splice variants affects vital parameters of the malignant breast cancer cell line MCF-7 in an opposing manner
Source: BMC Cancer. 2016 Aug 12;16:628. doi: 10.1186/s12885-016-2664-8 (PMC4983040; doi:10.1186/s12885-016-2664-8)
Supplement: Additional file 1: — Supplementary Figures. (PDF 1221 kb) [file 12885_2016_2664_MOESM1_ESM.pdf]

Supplementary Material

OVEREXPRESSION OF *KCNJ3* GENE SPLICE VARIANTS AFFECTS VITAL PARAMETERS OF THE MALIGNANT BREAST CANCER CELL LINE MCF-7 IN AN OPPOSING MANNER

REZANIA S.[1,*], KAMMERER S.[1,*], LI C.[1,*], STEINECKER-FROHNWIESER B.[1,*,#], GORISCHEK A.[1,*], DeVANEY T.T.J.[1,*], VERHEYEN S. [1,*,§], PASSEGGER C.A.[2], GHAFFARI TABRIZI-WIZSY N.[2], HACKL H.[3], PLATZER D.[1], ZARNANI A.H.[4], MALLE E.[5], JAHN S.W.[6], BAUERNHOFER T.[7,*] & SCHREIBMAYER W.[1,*,+].

[1]: Institute of Biophysics, Molecular Physiology Group, Medical University of Graz, Austria

[2]: Institute of Pathophysiology and Immunology, SFL Chicken CAM Laboratory, Medical University of Graz, Austria

[3]: Division of Bioinformatics, Biocenter, Medical University of Innsbruck, Austria

[4]: Nanobiotechnology Research Center, Avicenna Research Institute, Iran

[5]: Institute of Molecular Biology and Biochemistry, Medical University of Graz, Austria

[6]: Institute of Pathology, Medical University of Graz, Austria

[7]: Division of Oncology, Department of Internal Medicine, Medical University of Graz, Austria

[*]: Research Unit on Ion Channels and Cancer Biology, Medical University of Graz, Austria

[§]: present address: Institute of Human Genetics, Medical University of Graz, Austria

[#]: present address: Ludwig Boltzmann Department for Rehabilitation of Internal Diseases, Ludwig Boltzmann Cluster for Arthritis and Rehabilitation, Saalfelden, Austria.

[+]: corresponding author: Institute of Biophysics, Research Unit on Ion Channels and Cancer Biology, Harrachgasse 21/4, Medical University of Graz, Austria, Phone: +43 316 380 4155, Fax: +43 316 380 9660.

Supplementary Table 1.: statement of clonal cell lines that have been used for the current project.

MCF-7 derived cell line	*Clones*
MCF-7^{GIRK1a}	*C-T hG1a#2*<br>*C-T hG1a#3*<br>*N-T hG1a#20*<br>*N-T hG1a#21*
MCF-7^{GIRK1c}	*C-T hG1c#16*<br>*C-T hG1c#19*
MCF-7^{GIRK1d}	*N-T hG1d#1*<br>*N-T hG1d#7*
MCF-7^{eYFP}	*eYFP#8*<br>*eYFP#9*
MCF-7$^{G\beta\gamma}$	*G$\beta\gamma$#17*

Supplementary Figure 1: Subcellular localization of GIRK1 protein in stably transfected MCF-7 cells.

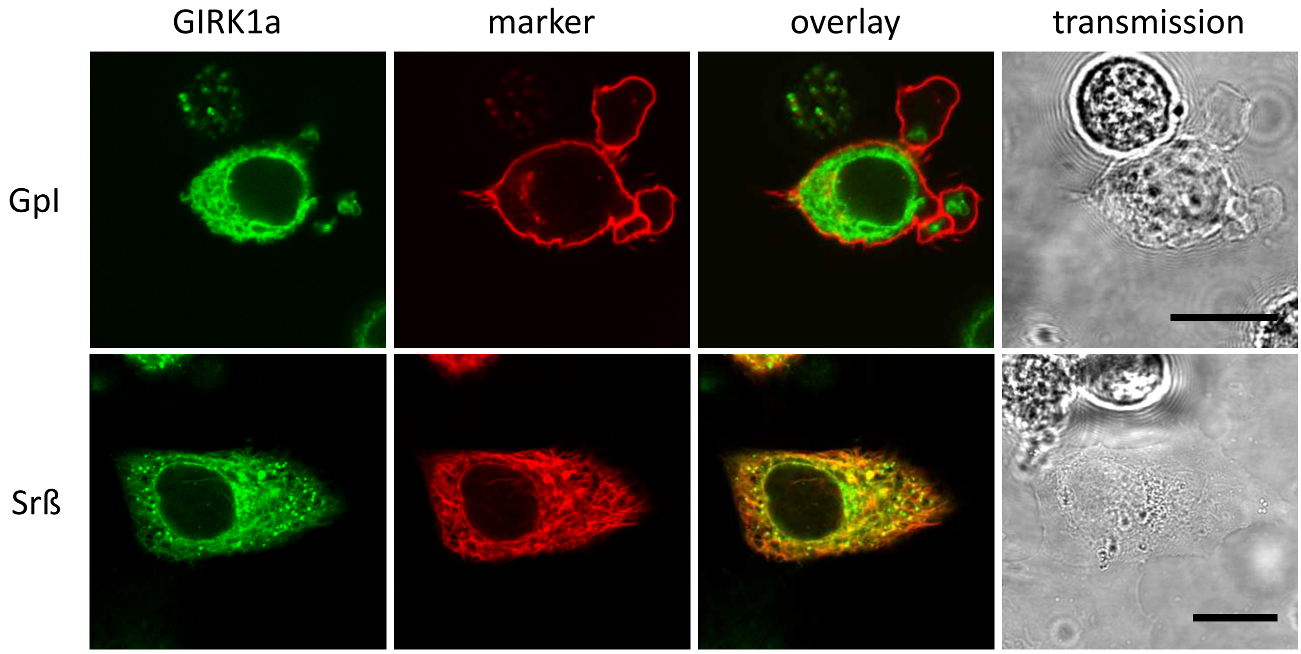

Horizontal sequences of images show identical cells. The sequence of channels (from left to right is: eYFP (*green*)/ eCFP (*red*)/ overlay / transmission. Scalebars: 15 µm in all images. *Upper panel:* subcellular localization of GIRK1a, labelled with eYFP at the N-T (GPI-eCFP was used as marker for lipid rafts in the plasma membrane). *Lower panel:* subcellular localization of GIRK1a, labelled with eYFP at the N-T (Srß-eCFP was used as ER marker).

Supplementary Figure 2: Overexpression of mRNA in individual clones of the stably transfected MCF-7 cells.

mRNA encoding GIRK1 was quantified by qPCR. Expression was normalized to the expression level in the MCF-7^{WT} cell line. *WT:* MCF-7^{WT}; *eYFP:* cell lines with stably integrated pEYFPN1 vector alone (#8; #9); *hG1a:* data derived from clones overexpressing GIRK1a fused at the C-T (#2; #3) and at the N-T (#20; #21) to eYFP. *hG1c:* data derived from clones overexpressing GIRK1c fused at the C-T to eYFP (#16; #19). *hG1d:* data derived from clones overexpressing GIRK1d fused at the N-T to eYFP (#1; #7). Mean values ± SEM were plotted (number of experiments is given in parenthesis above each bar).

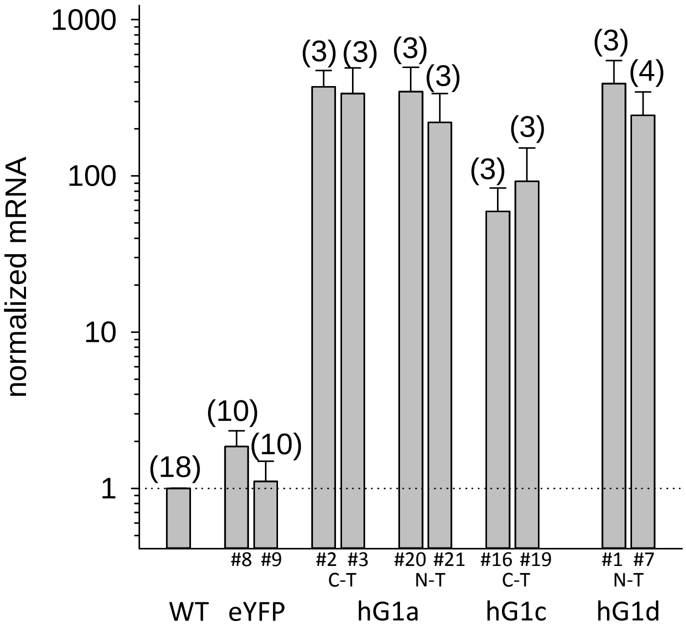

Supplementary Figure 3: Representative pairs of micrographs for chemoinvasion through Matrigel for all GIRK variants and controls tested.

Comprehensive view of fixated cells, stained with crystal violet, that managed to invade the Matrigel layer (10x magnification). The Scalebar corresponds to 100 µm for all images (variants are indicated right to the micrographs).

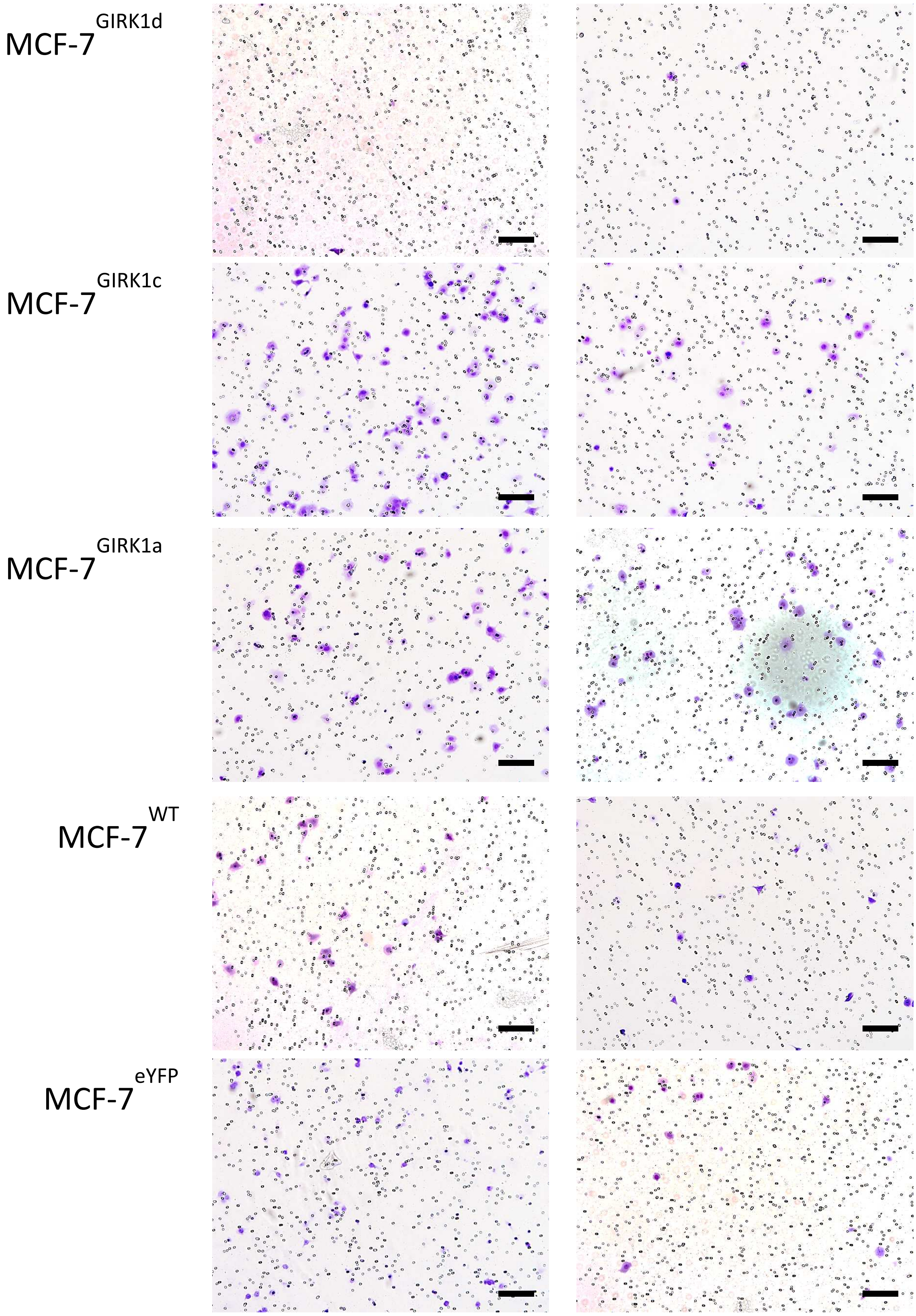

Supplementary Figure 4: Transient overexpression of GIRK1a increases both cellular velocities and motility coefficients in MCF-7^{WT} cells.

4A: Graphical representation of mean cellular velocities (v) for different experimental groups. *WT:* MCF-7^{WT} untreated; *eYFP:* MCF-7^{WT} transiently transfected with pEYFPN1 vector alone; *hG1a:* MCF-7^{WT} transiently transfected with GIRK1a/eYFP. The median value is represented by the black line within the box, box margins represent 75% and 25% percentiles, whiskers indicate 90% and 10% percentiles, values outside the 10%-90% interval are plotted individually (*crosses*). The grey line represents the mean value. The number of individual cells is given in parenthesis besides each box. Statistical significant differences between groups are indicated. hG1a differs from WT statistically significant at the $p < 0.05$ level. hG1a differs from eYFP statistically significant at the $p < 0.001$ level. Kruskal-Wallis one way analysis on ranks was used for analysis of statistical significance.

4B: Graphical representation of cellular motility coefficients (MC) for different experimental groups. *WT:* MCF-7^{WT} untreated; *eYFP:* MCF-7^{WT} transiently transfected with pEYFPN1 vector alone; *hG1a:* MCF-7^{WT} transiently transfected with GIRK1a/eYFP. The median value is represented by the black line within the box, box margins represent 75% and 25% percentiles, whiskers indicate 90% and 10% percentiles, values outside the 10%-90% interval are plotted individually as crosses. The grey line represents the mean value. The number of individual cells is given in parenthesis besides each box. Statistical significant differences between groups are indicated. hG1a differs from WT statistically significant at the $p < 0.05$ level. hG1a differs from eYFP statistically significant at the $p < 0.001$ level. Kruskal-Wallis one way analysis on ranks was used for analysis of statistical significance.

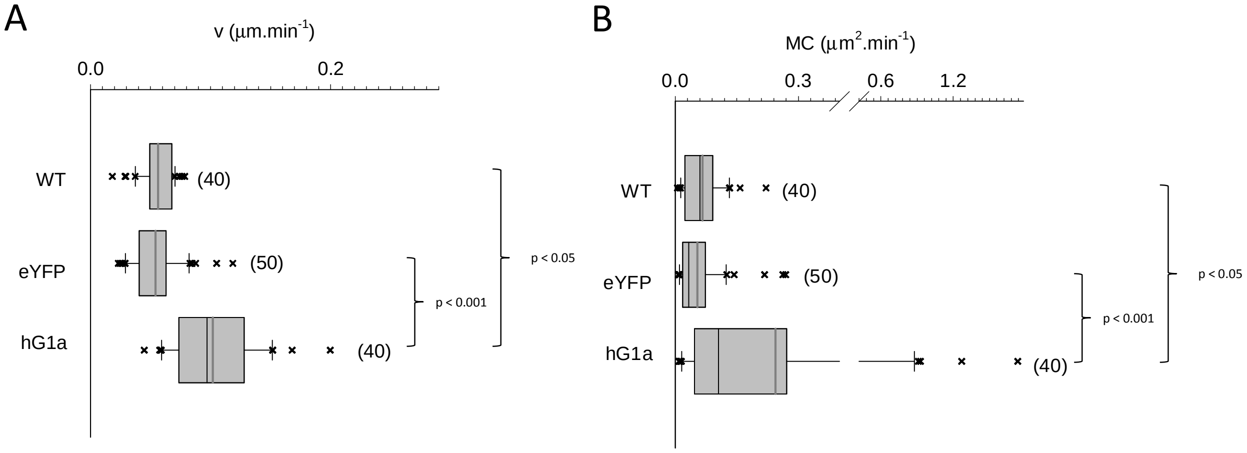

Supplementary Figure 5: Characterization of the MCF-7$^{G\beta\gamma}$ cell line and functional GIRK channel complex in the plasma membrane.

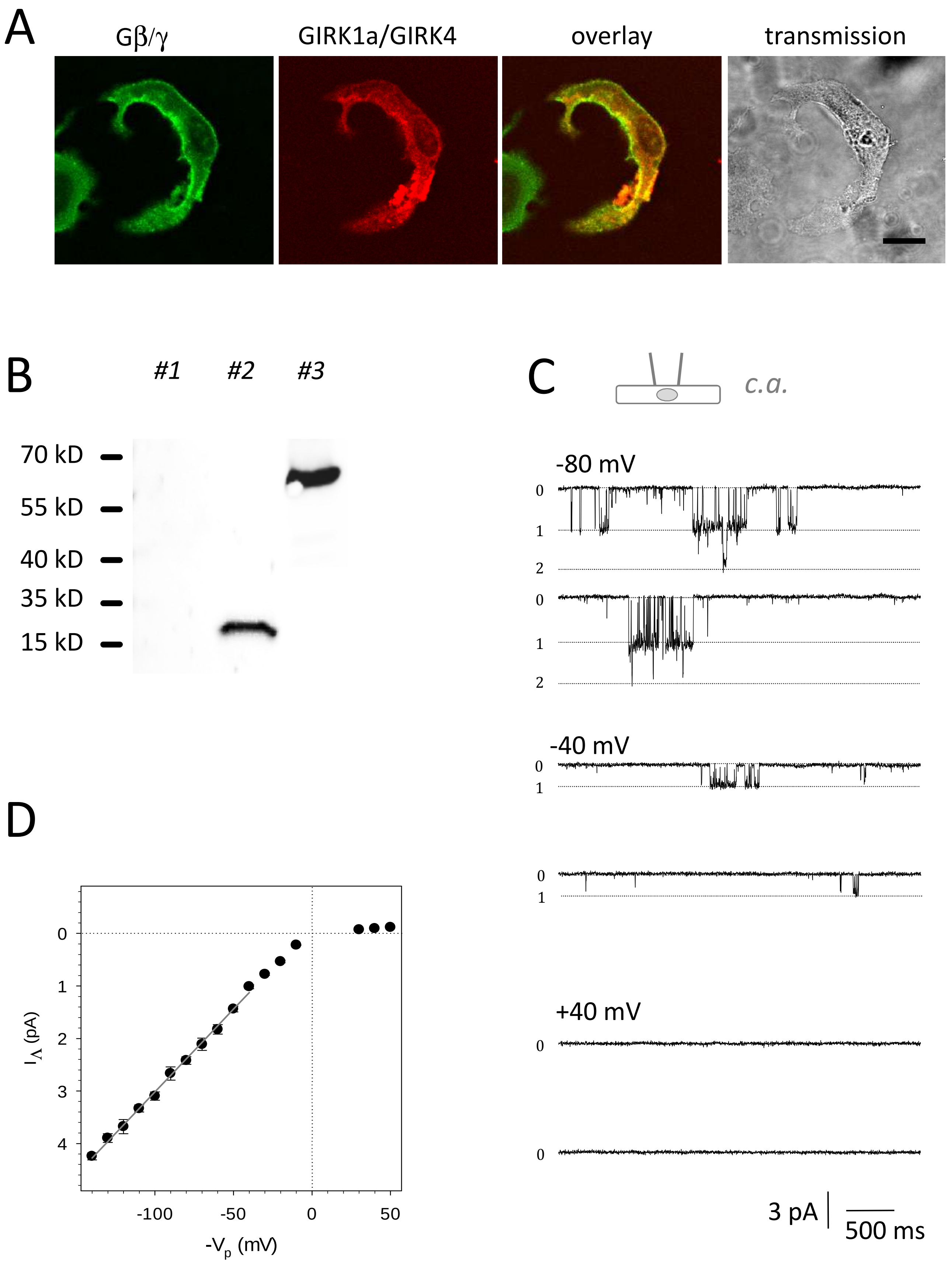

A MCF-7 based cell line stably expressing a biscistronic construct encoding a N-T Gβ/eYFP chimera and Gγ was produced ($MCF\text{-}7^{G\beta\gamma}$; see methods section). Successful expression was verified by cLSM and Western blot analysis (sFig6A&B). In order to screen for functional GIRK channels, single channel patch clamp recordings were performed in the cell attached mode on $MCF\text{-}7^{G\beta\gamma}$ and later, as the occurrence of functional channels was extremely rare, on $MCF\text{-}7^{G\beta\gamma}$ that were transiently transfected with GIRK1a and GIRK4, a combination known to result in functional GIRK channel activity [1].

5A: The sequence of channels (from left to right is: eYFP (*green*)/ eCFP (*red*)/ overlay / transmission. Scalebar: 15 μm. *Green:* Subcellular localization of Gβ, labelled with eYFP at the N-T. *Red:* subcellular localization of GIRK1a and GIRK4, labelled with eYFP at their N-T.

5B: Western blot (WB) analysis of Gβ/eYFP expression in the $MCF\text{-}7^{G\beta\gamma}$ cell line. WB was performed as described previously [1] with GFP antibody (Santa Cruz, USA; catalog No.: GFP (FL): sc-8334) diluted 1:200 (blocking 1 hour at room temperature, antibody incubation over night at 4°C). Cell lysates (40 μg protein/lane) were applied as follows: *#1:* $MCF\text{-}7^{WT}$; *#2:* $MCF\text{-}7^{eYFP}$, *#3:* $MCF\text{-}7^{G\beta\gamma}$

5C: Original current traces recorded from the $MCF\text{-}7^{G\beta\gamma * Girk1a*GIRK4}$ cell line in the cell attached recording configuration at different potentials.

5D: Single channel currents (I_Λ) vs. pipette potential ($-V_p$) for the record shown in 5C. Single channel conductance was G_Λ: 31.5 ± 0.6 (pS).

Supplementary Reference:

1. Wagner V, Stadelmeyer E, Riederer M, Regitnig P, Gorischek A, Devaney T, Schmidt K, Tritthart HA, Hirschberg K, Bauernhofer T *et al*: **Cloning and characterisation of GIRK1 variants resulting from alternative RNA editing of the KCNJ3 gene transcript in a human breast cancer cell line**. *J Cell Biochem* 2010, **110**(3):598-608.
